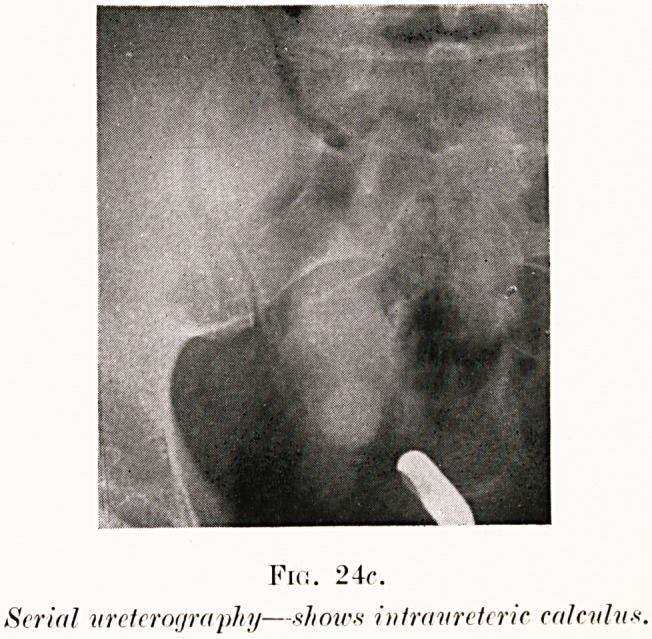# The Rôle of Pyelography in Renal Disorders
*A Paper read at a Meeting of the Bristol Medico-Chirurgical Society held at the University of Bristol on 9th January, 1929.


**Published:** 1929

**Authors:** A. Wilfrid Adams

**Affiliations:** Assistant Surgeon, Bristol Royal Infirmary and The Bristol Royal Hospital for Sick Children and Women


PLATE I.

Fig. 1.

*Healthy uretero-pyelograms.*

### THE ROLE OF PYELOGRAPHY IN RENAL DISORDERS.*

BY

A. Wilfrid Adams, M.S., F.R.C.S.,

*Assistant Surgeon, Bristol Royal Infirmary and The Bristol Royal Hospital for Sick Children and Women.*

---

As the race grows older it provides, with each generation, a crop of zealous workers concerned with cutting new and better paths to the summit of civilization. Sage elders, however, turn the pages of history and remark that most of our innovations are but rediscoveries. Despite this warning to the young enthusiast, I venture to suggest that in pyelography and the concomitant segregation of the urines of the kidneys, the diagnostic guesswork of the last century has been replaced by an almost exact science. It is an inestimable boon that it is possible, by means of the ingenious and delicate instruments recently invented, to peer into the complex recesses of a deep-seated organ like the kidney and, metaphorically, take test-tubes and culture media there as well as vision. This technique is all the more satisfactory in that, when the investigation is finished, the kidney is none the worse for the manipulations. It gives exact information of the hidden strongholds of a deeply-entrenched foe. The light of the science of to-day has triumphed over the darkness of the upper

* A Paper read at a Meeting of the Bristol Medico-Chirurgical Society held at the University of Bristol on 9th January, 1929.

urinary tract, even as the cystoscope first illumined the bladder in the last century. This has led to a revolution in urinary diagnosis which it is my purpose to portray.

Pyelography and allied processes may be regarded as equivalent to an exploration of the kidney by the closed method, justifying the title of "bloodless nephrotomy." It will appeal more to the medical practitioner, for whom diagnosis is of primary importance in treating disease, than to a suffering public whose only interest lies in the cure of their malady. On increasingly accurate diagnosis depends progress in the treatment of disease.

### TECHNIQUE.

*Preparation.*—Accurate diagnosis of a pyelogram often depends on fine distinctions. As shadows of bowel wall and contents, falling across the renal region, may confuse and distort the pictures, it is imperative to prepare the patient in this respect.

*Anæsthetic.*—A preliminary sedative, local urethral anæsthesia and a conscious patient are preferable to a general anæsthetic. For the beginner they are essential. The reason is that the best guide to good filling of the kidney is when the patient notices a warning discomfort in the side. It is thanks to this guide, also, that the danger of over-distention is prevented.

*Examination.*—The bladder cavity is inspected and abnormalities noted. Prior to threading the ureters with special long and slender catheters, their orifices and the character of the effluxes issuing from them are patiently observed. The catheter is then passed through a tunnel in the cystoscope till the operator sees the end projecting at the vesical eye of the cystoscope. A tribute needs to be paid to Albarran who invented the lever that next directs the end of the catheter into the ureter. The ureteric catheter is then passed up the duct. The ideal destination for it is when its tip is near the bottom or outlet of the renal cavity. Both kidneys are dealt with as a rule and with the double catheterizing cystoscope. Having introduced the catheters, and being satisfied they are working well, the surgeon may withdraw the cystoscope and relieve the patient of this discomfort. As a routine, however, I doubt the wisdom of, and do not do, this, as the catheters inevitably withdraw a certain distance and essential evidence may be lost. Preparation is made for radiography at this stage.

*Radiography.*—The need for careful placing of the patient so that kidney and X-ray plate correspond is apt to be forgotten. A plain X-ray of the whole tract antero-posteriorly, and laterally when necessary, is taken. Immediate development of these is helpful in revealing, by the level of the catheter tips, abnormally high or low kidneys. Without this knowledge the radiologist could not make the necessary adjustment in the position of his plate. Failure to recognize this possible error and guard against it has often rendered pyelography worthless. Then each kidney in turn is pyelographed as follows: Warmed opaque solution is run in, either by gravity (the best method for beginners) or from a syringe. The renal intake varies, the average being 5–8 c.c., but I have met with such extremes as 3–15 c.c. respectively in health. This makes it very difficult to foretell what is the approximate quantity for any patient. As soon as pain is felt or the average amount has entered the kidney, the patient is instructed to hold his breath and keep quite still. The radiogram is taken at once and developed, as it may have to be repeated. The opaque medium is then withdrawn. Ensuing lavage is of doubtful merit. The catheter and cystoscope are now removed. If delayed emptying from pyeloureteric obstruction is suspected, then the catheter is withdrawn without the preliminary aspiration of the fluid, which is left for the kidney to expel. The time this takes is revealed by a series of pyelograms taken at intervals until the shadow has faded.

*After-care and Complications.*—The patient is immediately provided with hot bottles, morphia (s.o.s.) and copious drinks. Pain varies from a negligible degree to genuine renal colic. It may last three or four days. Vomiting occurs occasionally, and the patient sometimes suffers from shock. Hæmaturia, microscopic or slightly visible, is common; in a few cases it is considerable.

### THE SCOPE OF PYELOGRAPHY.

A preliminary analysis of the evidence the picture affords may facilitate diagnosis of actual cases. (See chart.)

*The Normal Pyelogram.*—This tells us the number, site and shape of the kidneys and ureters (see Fig. 1). It reveals the negative evidence of the presence of a healthy kidney, which is information of priceless value in the differentiation of obscure abdominal pains and swellings. All the more precious is such information since, in a general search of the peritoneal cavity at abdominal exploration, the kidney is not open to easy examination. Mere palpation may leave one

### PYELOGRAPHIC CHART

ignorant of early infectious or neoplastic disorders. Further, the sure knowledge of one healthy organ which it affords gives the surgeon complete confidence in dealing with the fellow-kidney when diseased. There is a natural difficulty in reading healthy pyelograms, for, although externally kidneys maintain their well-known form, their interior cavities are as protean in form as are men's faces. This makes detection of early disorders of structure only possible after much experience in the reading of the radiograms. Added to this natural variation there is the still further surprise that the two healthy kidneys of the same patient are asymmetrical and often grossly different. No wonder the question is often asked, " What is a normal pyelogram ? "

In the range of normal pyelography great variety of form must be expected, just as in the features of healthy faces. Ill-health is deduced from altered expression rather than from the form of the face. As in faces, so it is in kidneys. Thus, in the diagnosis of early hydronephrosis the observer pays more attention to a bloated expression in the pyelogram and rather disregards the directions and number of the calyces.

It is worth repeating that for pyelography to establish, with scientific certainty, that the kidneys are present and normal, is one of the most valuable and common services it renders.

*Abnormalities.*—Among the abnormalities it depicts, some of which are shown in the chart, are :—

*A.*—Congenital—solitary, fused, ectopic and polycystic types.

*B.*—Acquired—(*a*) Hydronephrosis, general and local, primary (due to kinked outlet or non-dependent drainage), or secondary (due to calculi, tumours, etc.). (*b*) Irregularity and roughening of the pelvis. (*c*) Filling defects, " bays," etc., of the pelvic walls. (*d*) Calculi—defining the exact nature and site. Such pictorial abnormalities have their correlation in disease.

### ILLUSTRATIVE CASES.

My next step is to illustrate how pyelography has helped in the elucidation of the underlying disorders which caused the clinical symptoms of my cases, and so laid the sure foundation for sound treatment. Cases come with clinical labels ; but these are crude categories, and we have to diagnose accurately by information obtained from all corners of the urinary system.

This article is based on a total of 129 cases, of which 111 were hospital cases and 18 private cases. Of these 53 were males, 71 females, and 5 children. The age limits were from 4 to 77 years. I have divided the cases into clinical groups according to the patient's dominant symptom. Naturally some of the groups overlap, inasmuch as some come into two groups, *e.g.* renal colic and hæmaturia. The figures shown belong to illustrative cases taken from the seven main groups which are as follows :—

I. Renal colic—30 cases.
II. Hæmaturia—17 cases.
III. Dysuria—24 cases.
IV. Fistula—3 cases.
V. Abdominal aches and pains—43 cases.
VI. Swellings in the hypochondrium—10 cases.
VII. Calculus—16 cases.

### PLATE II.

Fig. 2.—*Hydronephrosis (right).*

Fig. 3.—*Hydronephrosis (right).*

Fig. 4.—*Hemi-hydronephrosis (right).*

Fig. 5.—*Hydronephrosis (left).*

Fig. 6.—*Ureteric obstruction (right). Due to acute kink.*

Fig. 7.—*Hydronephrosis (right).*

PLATE III.

Fig. 8*a*. *Hypernephroma of right kidney.* Fig. 8*b*.

Fig. 8*c*.—*Pyelogram of removed kidney showing relation of stunted calyces to tumour.*

Fig. 9.—*Hypernephroma (left).*

Fig. 10*a*.—*Tumour with pelvic growth in right kidney.*

Fig. 10*b*.—*Pyelogram of removed kidney with filling defect in pelvis.*

PLATE IV.

FIG. 11.—*Hydronephrosis (right).*

FIG. 12*a*.—*Right pyonephrosis (tuberculous).*

FIG. 12*b*.—*Right renal tuberculosis.*

FIG. 13.—*Closed hydronephrosis (right). Normal pyelogram (left).*

FIG. 14.—*Blocked ureteric outlet (right) associated with renal fistula.*

### PLATE V.

Fig. 15.—*Horseshoe kidney.*

Fig. 16.—*Primitive tubular pelvis (left).*

Fig. 17.—*Bilateral polycystic disease.*

Fig. 18*a*.
*Carbuncle invading upper half of kidney.*

Fig. 18*b*.

*Pyelograms of both kidneys in same case.*

PLATE VI.

FIG. 19.—*Large pyonephrosis—ureter kinked.*

FIG. 20.—*Pyonephrosis—kinked ureter.*

FIG. 21.—*Perinephric abscess—deformed renal pelvis.*

FIG. 22.—*Healthy pyelogram of suspected kidney.*

PLATE VII.

FIG. 23.

*Normal pyelogram—position of kidney in relation to overlying retroperitoneal tumour.*

*Class I.*—Renal colic. Figs. 2–9.

Fig. 2. E. L.—Attack of renal colic and dysuria accompanied by pyrexia. Operation refused. Patient untraced. Pyelogram shows dilated pelvis, while kinked ureteric outlet suggests causal aberrant artery to lower pole of the kidney.

Fig. 3. A. C.—Symptoms and pyelograms similar to E. L. Causal aberrant artery found and divided at operation. Two years later reports cured.

Fig. 4. P. F.—Symptoms as E. L., but with hæmaturia. Comparison of pyelograms (bilateral) shows bi-partite pelvis, the lower half on the right side being dilated. Operation—pyelotomy and dilatation of the outlet. Reports cured.

Fig. 5. N. H.—Left renal colic. Pyelogram shows dropped kidney and typical pelvic type of hydronephrosis resulting. Pyeloplasty and nephropexy have cured.

Fig. 6. R. P.—Attacks of right renal colic for ten years and heavy hæmaturia. Ureteropyelogram shows a rare acute angulation of ureter. Operation declined.

Fig. 7. R. T.—Right renal colic. Pelvis dilated to 55 c.cs. Operation revealed causal aberrant artery to lower pole. Function of kidney justified conservative measures and obstruction was circumvented by lateral pyeloureteric anastomosis. Probably may yet need nephrectomy.

Figs. 8 (*a–c*). N. M.—Five weeks' history of severe renal colic (right), but only one slight hæmaturia. Right pyelogram shows " bay " filling defect and abortive calyces at arrow (see chart). Operation (2nd January, 1929) revealed early hypernephroma at position corresponding to pyelographic defect. Pyelography was repeated on the removed kidney for confirmation as seen in Fig. 8*c*.

Fig. 9. J. W.—Recent severe renal colic (bilateral) with hæmaturia. Pyelogram revealed " bay " filling defect. Operation (17th February, 1926) confirmed early hypernephroma at corresponding site in left kidney. Nephrectomy and insertion of radium. Recurrence in spring, 1928.

*Class II.*—Hæmaturia. Figs. 10, 11.

Figs. 10 (*a–b*). C. D.—Heavy hæmaturia with "clot" retention. This prevented preoperative pyelography. At operation kidney was removed containing growth with pelvic nodule. Opaque filling of specimen after removal shows area of negative density at site of pelvic tumour. Dr. A. D. Fraser reports "hypernephroma," as with Figs. 8 and 9. Died five days later.

Fig. 11. P. S. (aged 4).—Heavy hæmaturia on two consecutive week-ends. Early dilation of pelvis of right kidney is seen by pyelography and excretion from right side was deficient. Aberrant vessel divided at operation.

*Class III.*—Dysuria. Figs. 12, 13.

Figs. 12 (*a–b*). M. S.—Frequent and painful micturition for a few months. Pyelography revealed cavernous ulceration of right kidney and unaltered left side. Specimen (Fig. 12 *b*) was removed (October, 1928), despite presence of tuberculous organisms and pus in urine of both sides. She has done remarkably well.

Fig. 13. F. C.—Had cystitis with tubercle bacilli in urine in 1914, but recent trouble was partial incontinence of urine and right-sided pain. Right ureter was completely stenosed, preventing pyelography of that side, and no tubercle bacilli were found in the urine. Operation revealed closed hydronephrosis.

*Class IV.*—Fistula. Fig. 14.

Fig. 14. B. W.—Recurrent attacks of acute pyonephrosis with fistula in loin at site of two former nephrotomies. Right renal outlet blocked (? by stone, shown at catheter tip). Pyonephrectomy performed, as pyelogram of left kidney showed a healthy structure.

*Class V.*—Abdominal aches and pains. Figs. 1, 15–18.

Fig. 1. H. W.—Chronic loin ache proved non-renal by pyelography.

Fig. 15. C. F.—Pain in the right loin. Operation revealed horseshoe kidney as likely explanation of the convergence of the lower poles of pyelographic shadows.

Fig. 16. S. C.—Symptoms of left-sided pyelitis present. Primitive tubular state of pelvis found and anomalous structure confirmed in the removed kidney.

Fig. 17. M. H.—Admitted as suspected acute appendicitis of one day's duration. Operation revealed suppurative B. coli infection of cysts in enlarged right kidney. Pyelography shows irregularity of calyces, which are stumpy and of mushroom type.

PLATE VIII.

FIG. 24*a*.

*Right hydronephrosis—suspicious presacral shadow.*

FIG. 24*b*.

*Ureterogram of mega-ureter.*

FIG. 24*c*.

*Serial ureterography—shows intraureteric calculus.*

Figs. 18 (*a–b*). S. E.—Suffered right-sided pain for many months, variably diagnosed as pleurisy and pyelitis. Urine was sterile. Pyelography shows slight swelling of calyces suggesting early hydronephrosis. Kidney removed at operation had disorganized upper half riddled with necrotic nests of staphylococcal infection, *i.e.* carbuncle of kidney.

*Class VI.*—Swellings of the hypochondrium. Figs. 19–23.

Swellings of a dubious nature in the upper lateral abdomen form a distinct clinical group and afford frequent enigmas. For such a clinical conundrum the inclusive title of " hypochondrioma " is suggested. Figs. 19–23 are the pyelographic findings in five typical cases of this category.

Fig. 19. S. A.—The doctor called me to see this as a case of perforated gastric ulcer. It seemed that her trouble might be due to sudden complete blocking of a pyonephrosis, and pyelography clinched the diagnosis, for the ureter is seen kinked at the junction with the kidney. Nephrectomy has cured her.

Fig. 20. D. P.—Recurrent attacks of abdominal pain was the history of this case. Pyelography revealed a hydronephrosis, and the kink of the ureter to which this was attributable. Nephrectomy was curative.

Fig. 21. M. L.—This patient had a fixed tumour in the left abdomen associated with wasting and fever. Neoplasm and sepsis seemed equally possible clinically. Right pyelogram proved to be normal, while the left showed only two calyces filled. Operation showed this to be due to a perinephric abscess and drainage proved curative.

Fig. 22. A. S.—Large left " hypochondrioma " proved by pyelogram not to be renal. At a subsequent operation it was found to be splenomegaly due to Banti's disease.

Figs. 23. W. S.—Appeared healthy, but complained of recent attacks of right abdominal pain. A mobile mass was found in the right loin having the characters of a large renal swelling. Thanks to the pyelogram, which excluded the possibility of a renal origin, a diagnostic error and incorrect incision were avoided. A tumour defying removal and weighing $4\frac{1}{2}$ lbs. (1,850 grammes) was revealed at operation. It extended from the right loin to the left behind the lesser sac. Hemorrhage was profuse and resulted in death shortly after. Dr. Fraser reports it a fibroma.

*Class VII.*—Calculi. Fig. 24.

Fig. 24 (*a–c*). H. W.—This was a case of renal colic. The right ureteric catheter is seen to be acutely looped on itself, and had the radiologist not included the lowest level of the ureter in the skiagram, the large right hydronephrosis (30 c.c.) might well have been attributed to this twist. The cause of the trouble was, however, the calculus seen near the bladder. There was, at first, some doubt as to whether this shadow was within the ureter, since it was slightly off the line of the opaque catheter. The intraureteric position of the calculus was established by the further injection of opaque solution above it. Serial ureterograms were taken (Fig. 24, *b* and *c*). These showed the ureter dilated to the size of small intestine and the stone actually included in its shadow. This explained the anomalous twist of the ureteric catheter to be due, not to what appeared an incredible twist of the ureter, but to its looping in the spacious dilatation of the duct. Extraperitoneal operation allowed easy removal of the stone.

I wish to acknowledge my indebtedness to Dr. F. J. A. Mayes and his staff in the radiological department of the Bristol Royal Infirmary, on whose unstinted help the success of this work so largely depends.

## Figures and Tables

**Fig. 1. f1:**
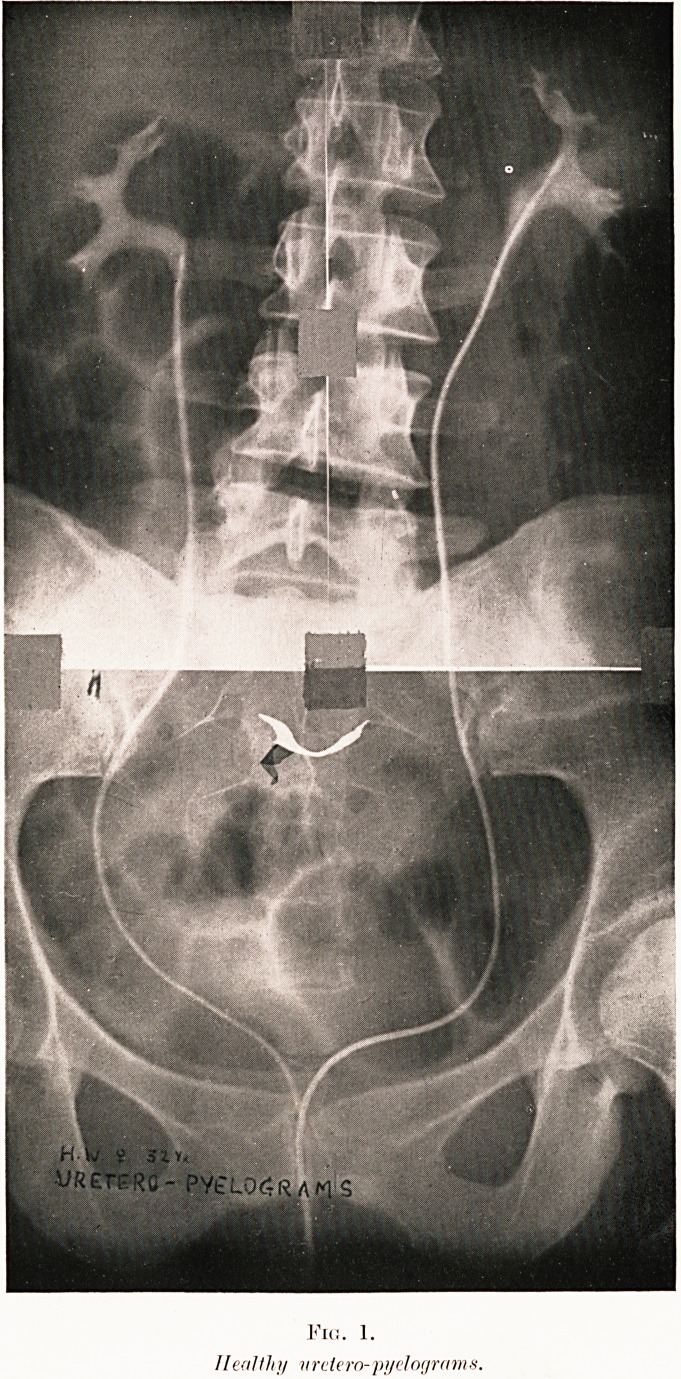


**Figure f2:**
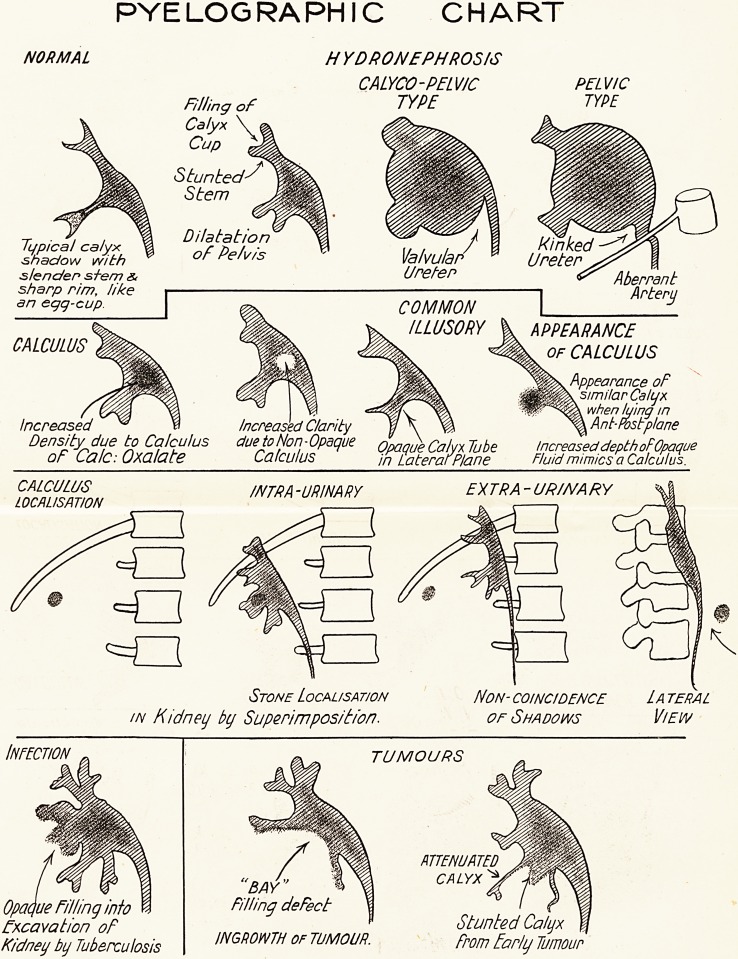


**Fig. 2. f3:**
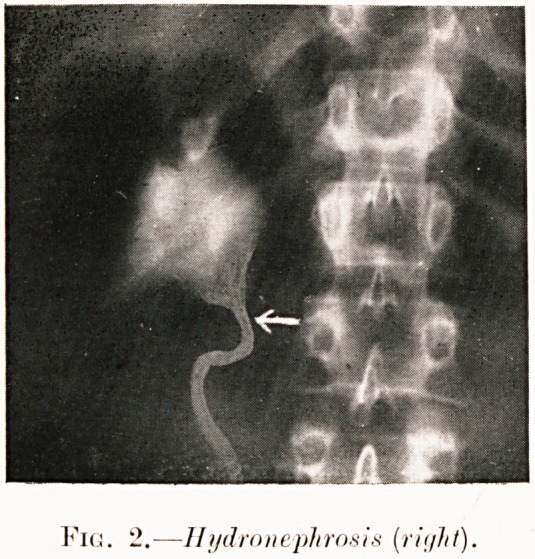


**Fig. 3. f4:**
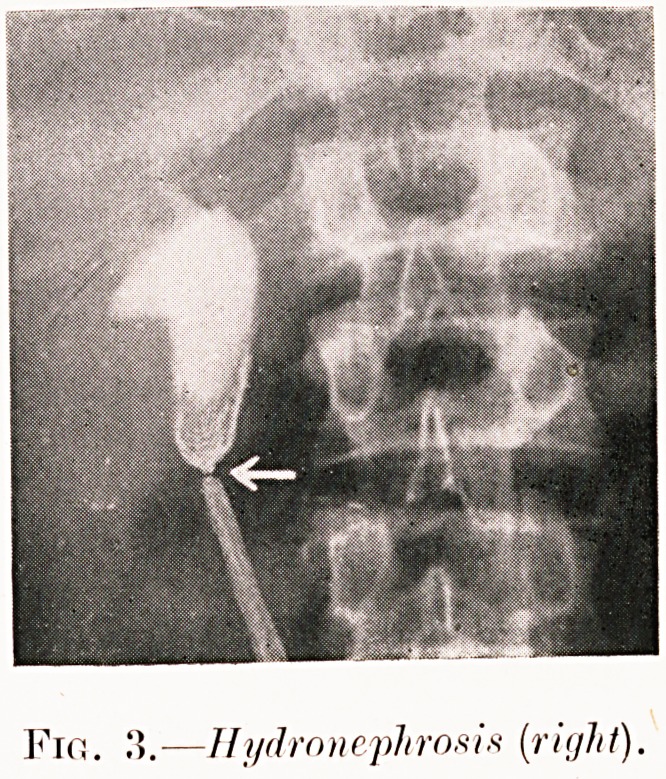


**Fig. 4. f5:**
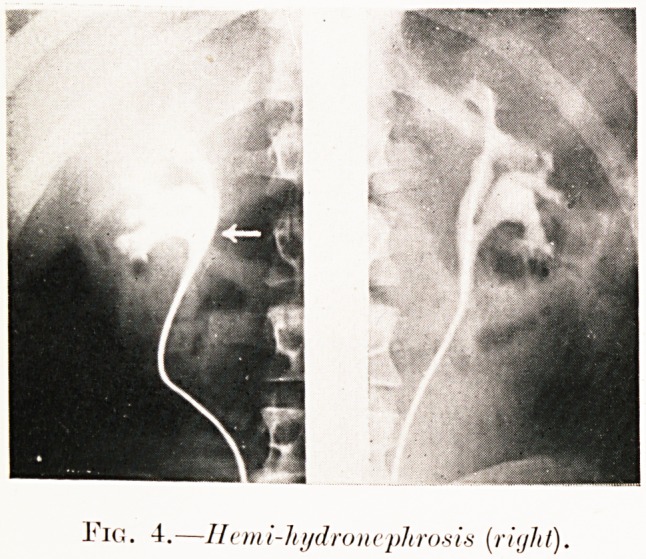


**Fig. 5. f6:**
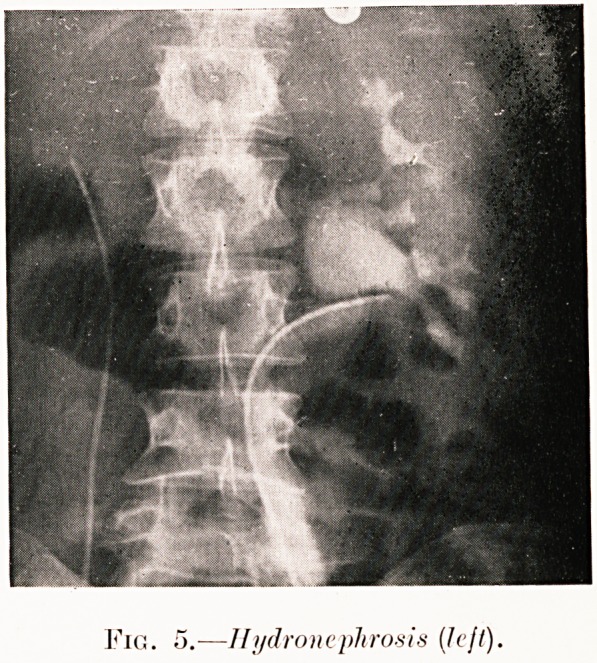


**Fig. 6. f7:**
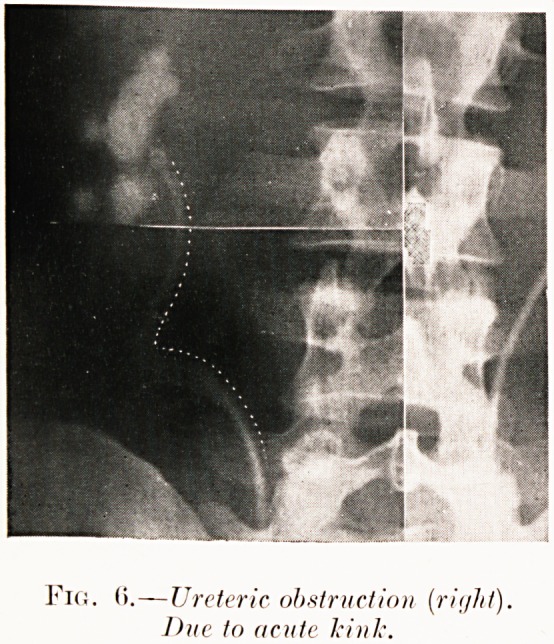


**Fig. 7. f8:**
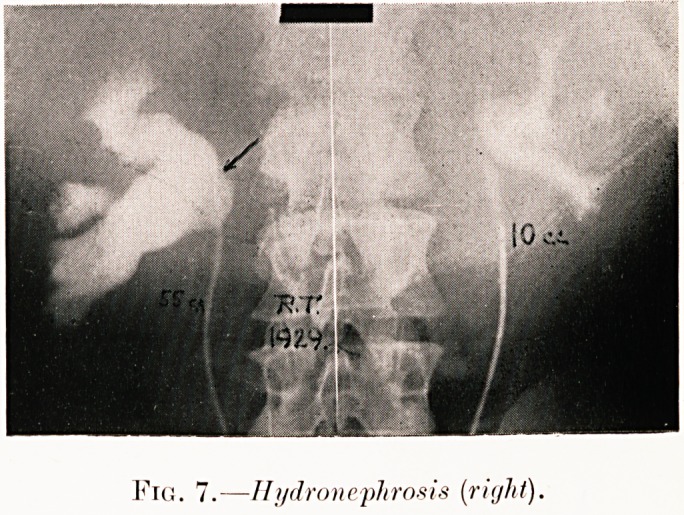


**Fig. 8a. Fig. 8b. f9:**
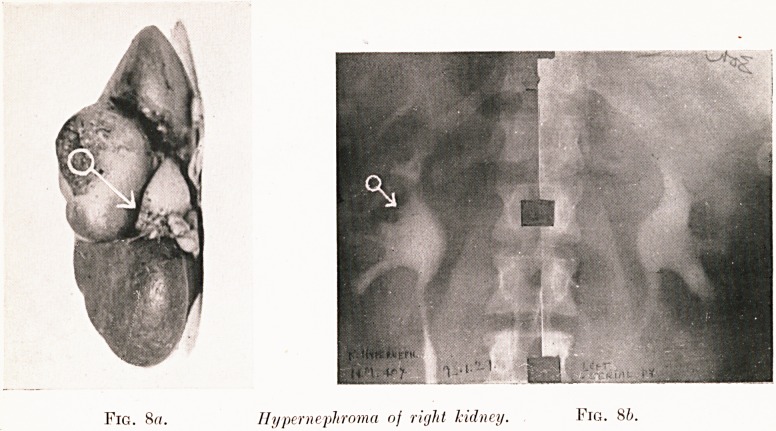


**Fig. 8c. f10:**
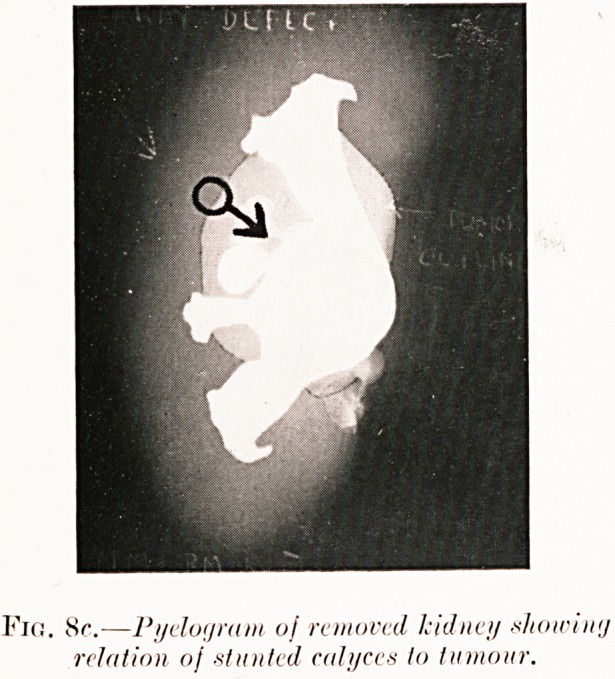


**Fig. 9. f11:**
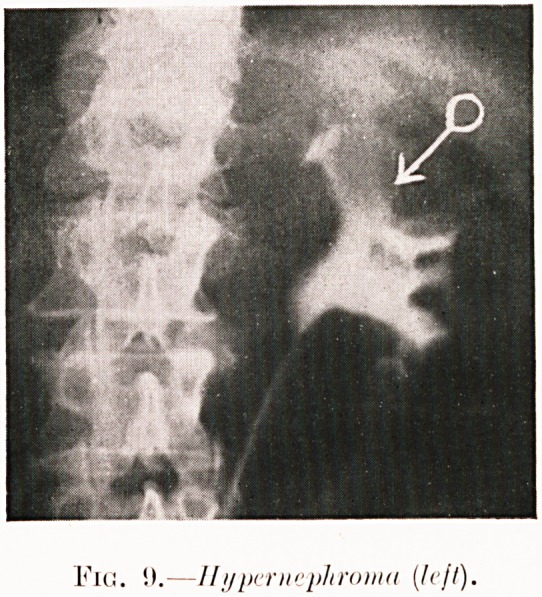


**Fig. 10a. f12:**
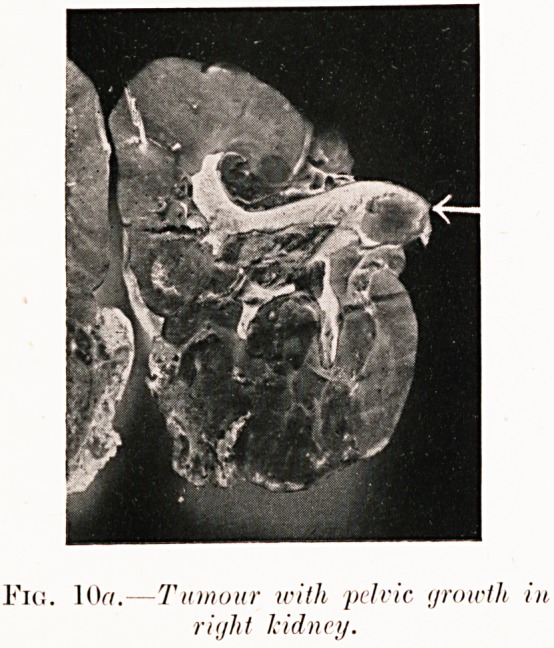


**Fig. 10b. f13:**
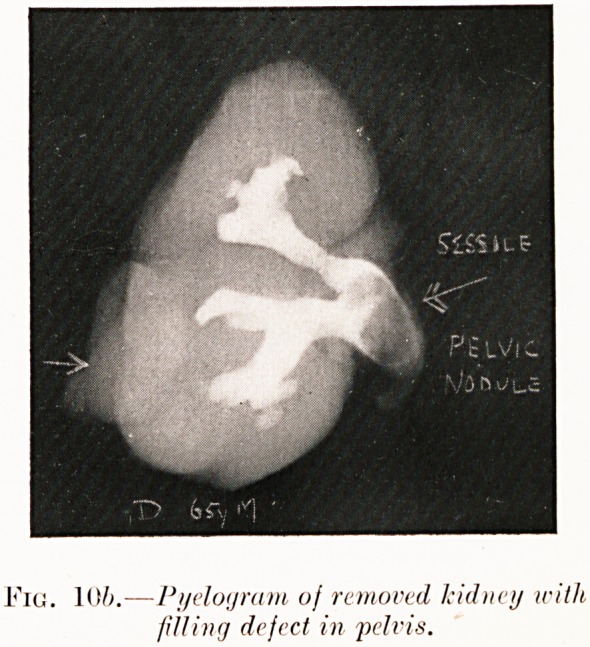


**Fig. 11. f14:**
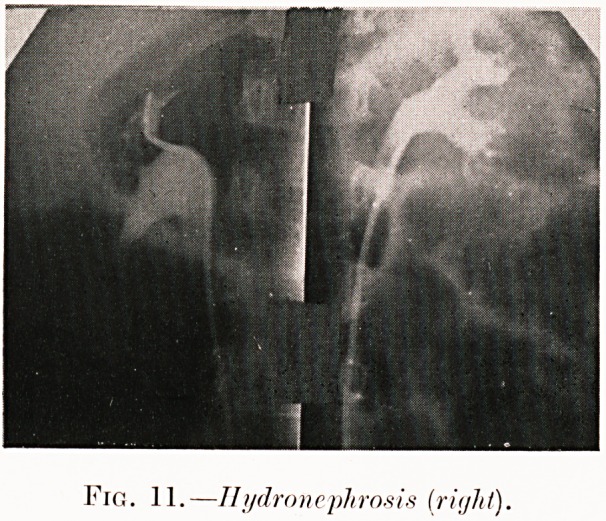


**Fig. 12a. f15:**
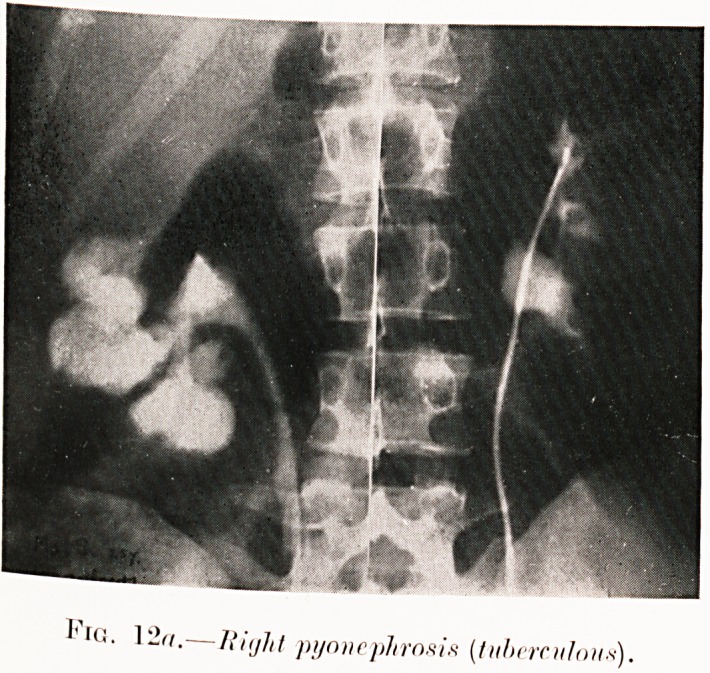


**Fig. 12b. f16:**
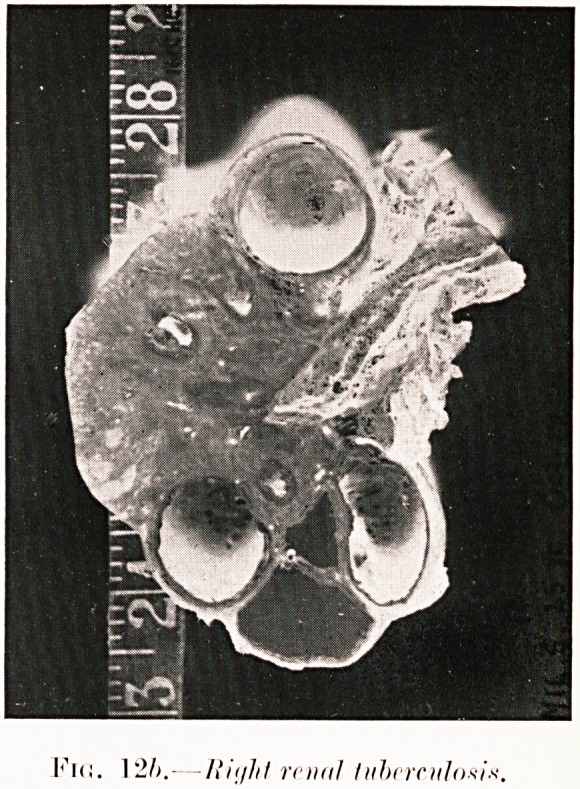


**Fig. 13. f17:**
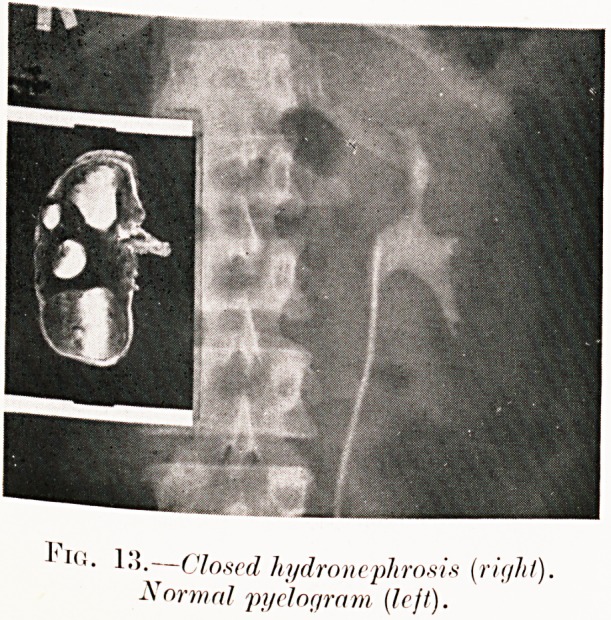


**Fig. 14. f18:**
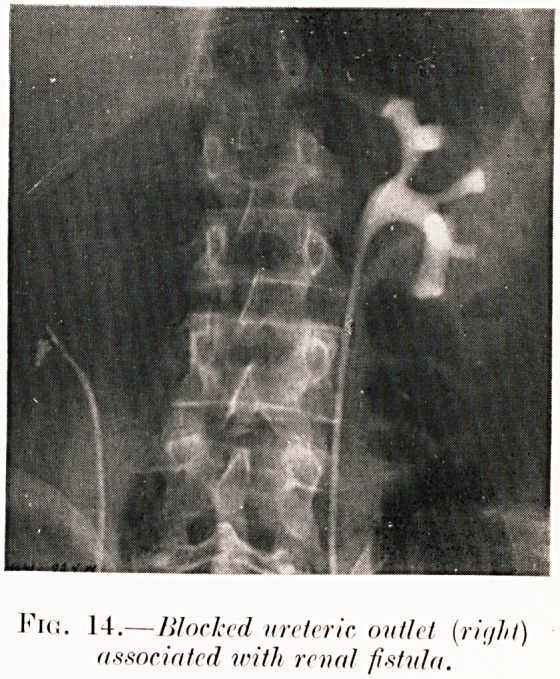


**Fig. 15. f19:**
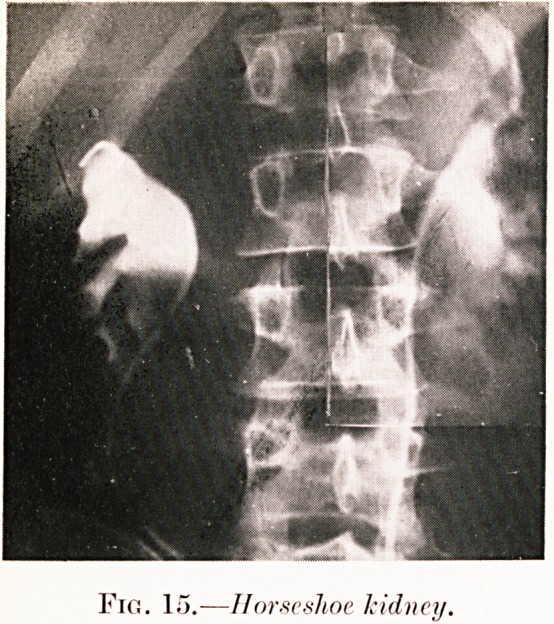


**Fig. 16. f20:**
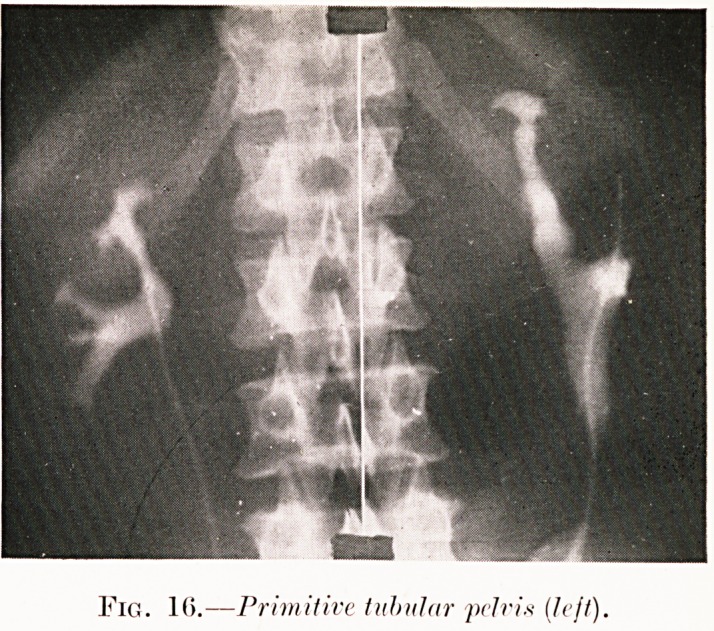


**Fig. 17. f21:**
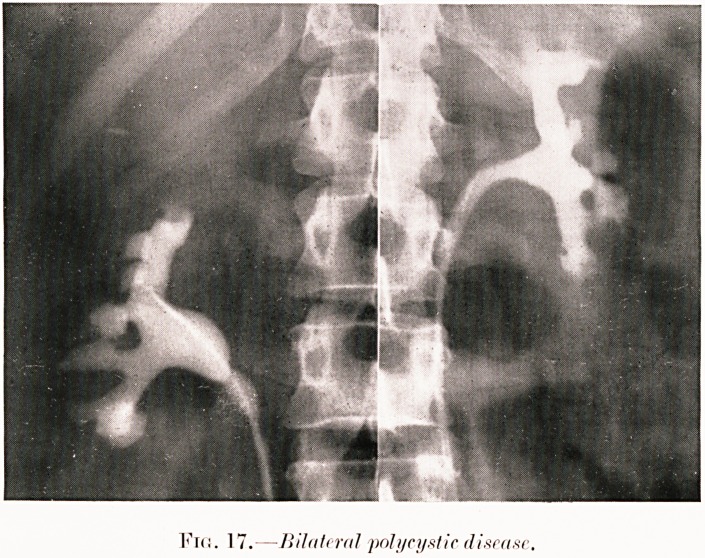


**Fig. 18a. f22:**
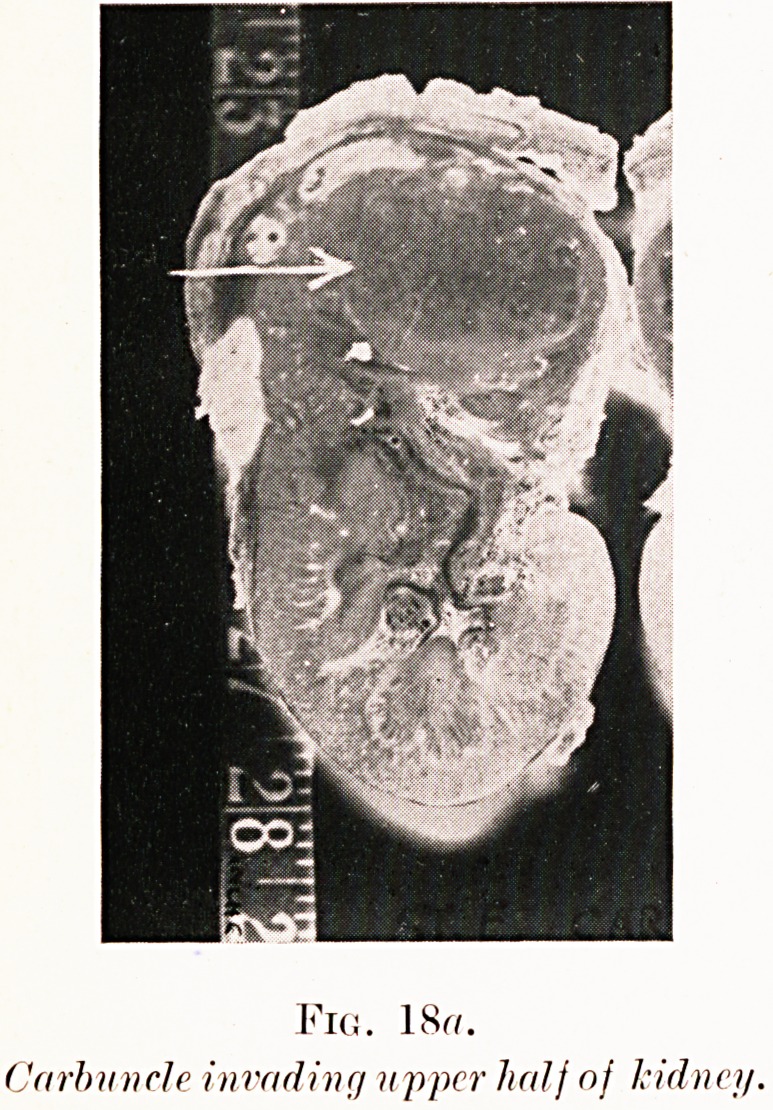


**Fig. 18b. f23:**
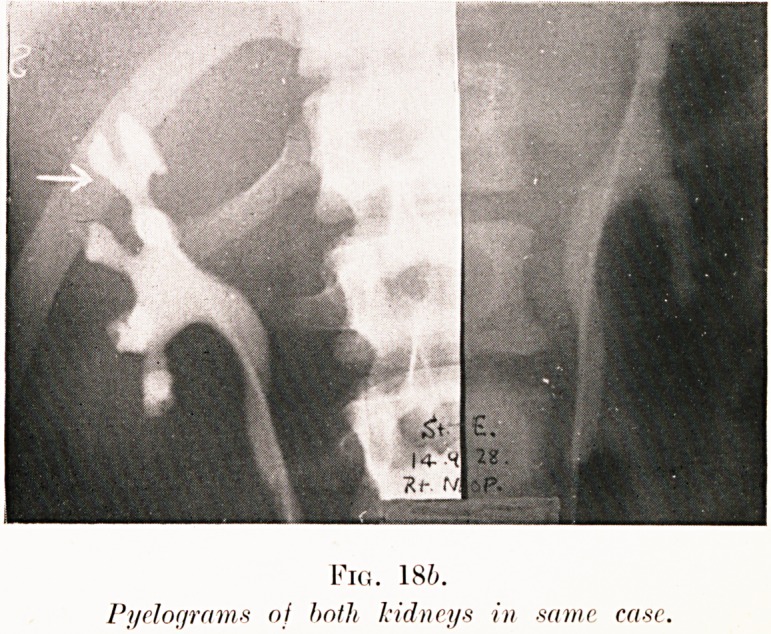


**Fig. 19. f24:**
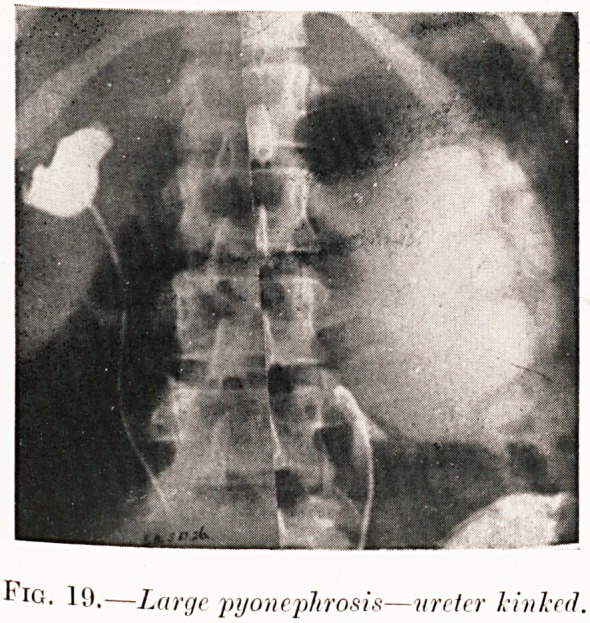


**Fig. 20. f25:**
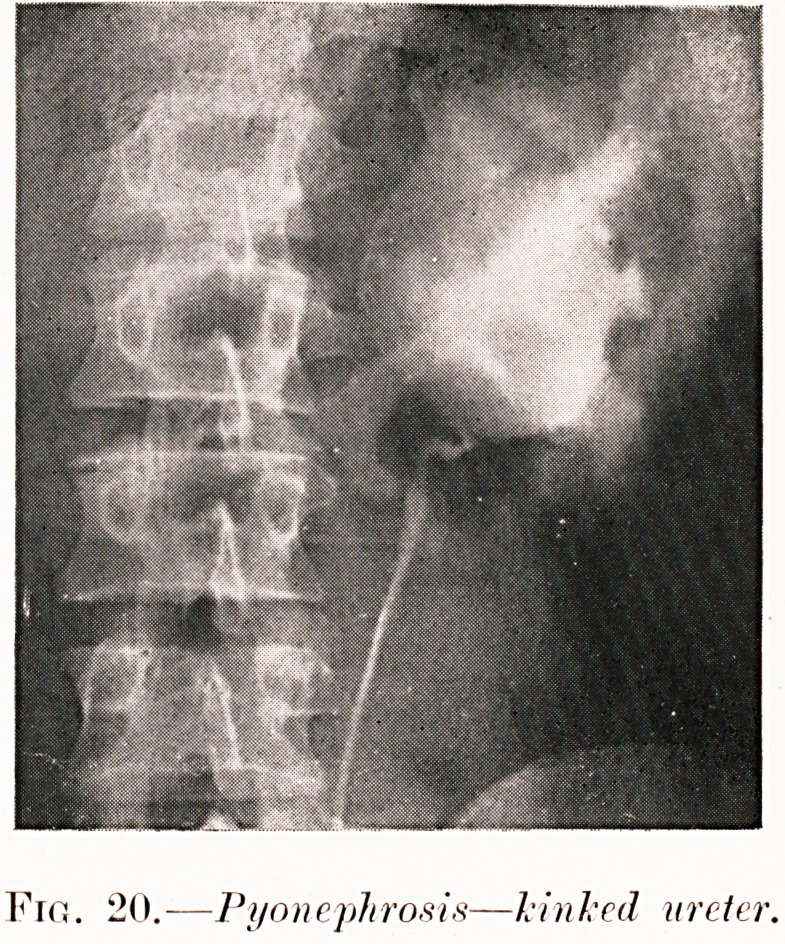


**Fig. 21. f26:**
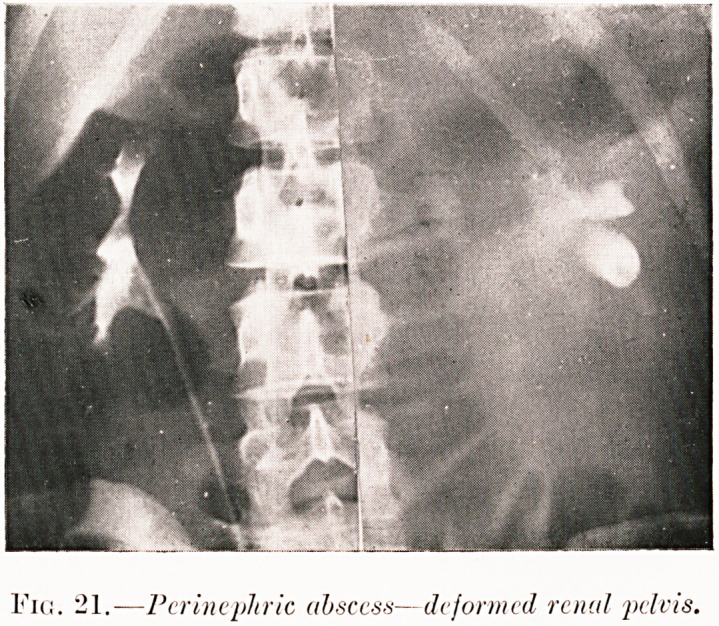


**Fig. 22. f27:**
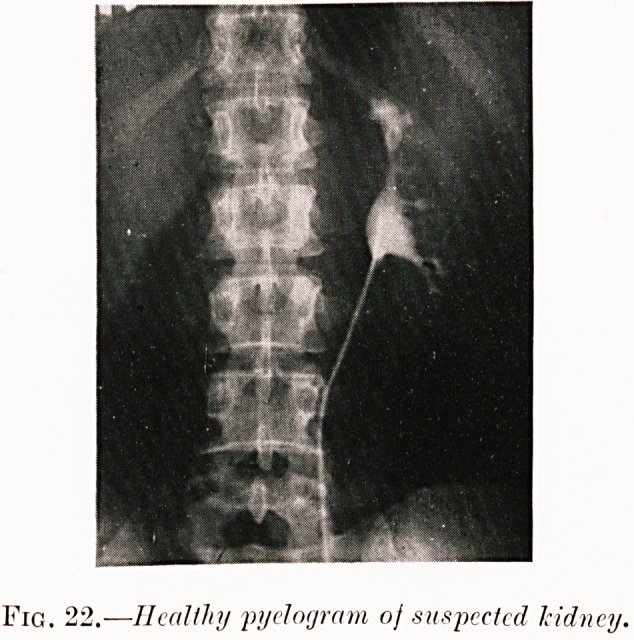


**Fig. 23. f28:**
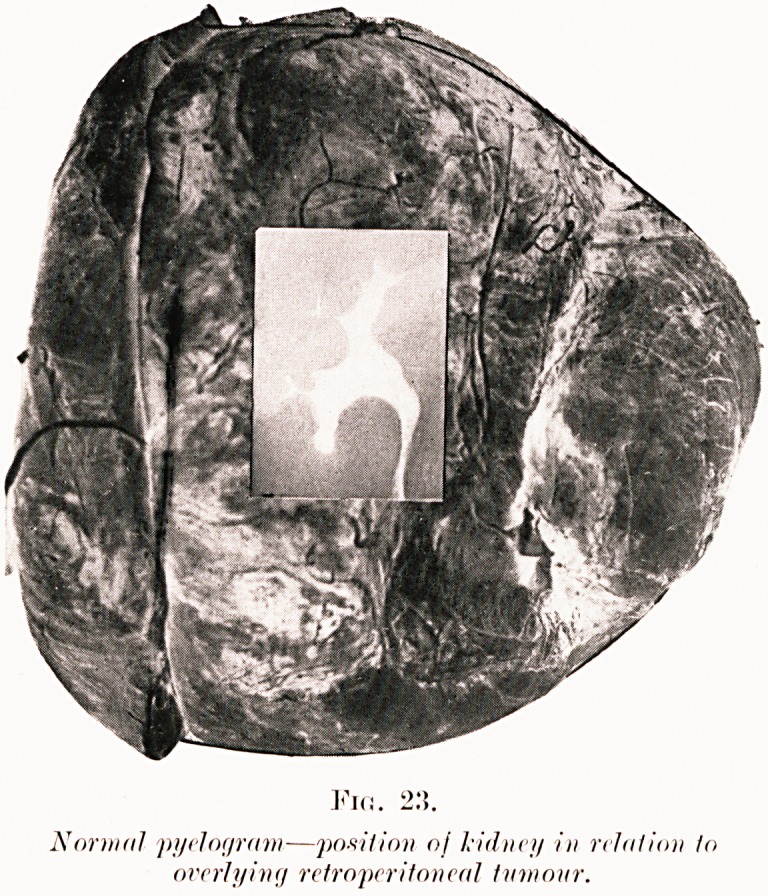


**Fig. 24a. f29:**
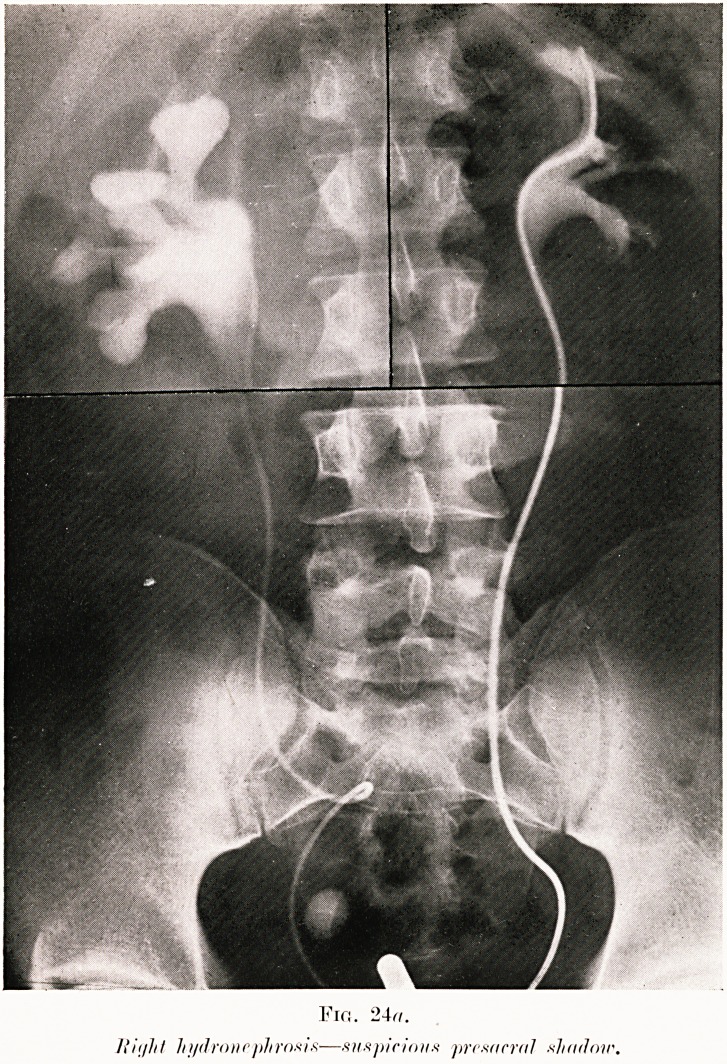


**Fig. 24b. f30:**
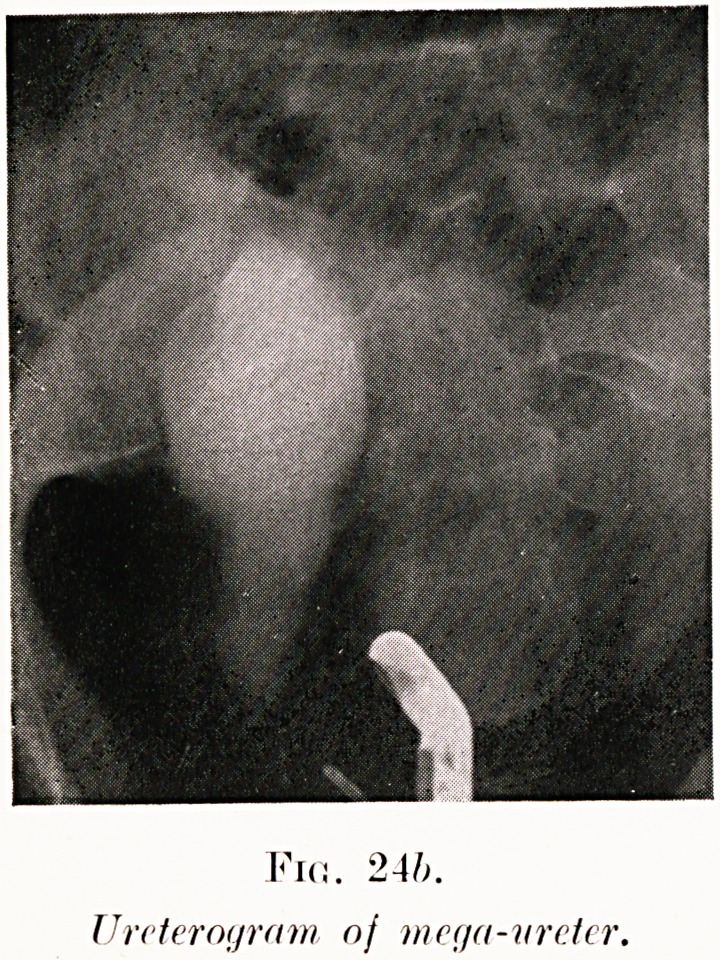


**Fig. 24c. f31:**